# Focus on climate change and plant abiotic stress biology

**DOI:** 10.1093/plcell/koac329

**Published:** 2022-11-15

**Authors:** Nancy A Eckardt, Sean Cutler, Thomas E Juenger, Amy Marshall-Colon, Michael Udvardi, Paul E Verslues

**Affiliations:** Senior Features Editor, The Plant Cell, American Society of Plant Biologists, USA; Guest Editor, The Plant Cell and Institute for Integrative Genome Biology, University of California, Riverside, California 92521, USA; Guest Editor, The Plant Cell and Department of Integrative Biology, University of Texas, Austin, Texas 78712, USA; Guest Editor, The Plant Cell and University of Illinois at Urbana-Champaign, Urbana, Illinois 61801, USA; Reviewing Editor, The Plant Cell and University of Queensland, St Lucia QLD 4072, Australia; Senior Editor, The Plant Cell and Institute of Plant and Microbial Biology, Academia Sinica, Taipei, Taiwan 11529

In many regions of the world, climate change is leading to increased exposure to abiotic stresses for plants as well as humans and other animals. The most obvious effects are warmer temperatures, more frequent episodes of extreme heat, increased drought conditions, and desertification, and in some regions, more frequent and extreme storms and flooding. This focus issue of *The Plant Cell* aims to spotlight research focusing on the biology of plant response to abiotic stresses, as well as more applied research aimed at mitigating the negative effects of climate change.

Drought stress is a primary concern in crop production and plant ecophysiology as it can cause significant reductions in crop yield and restrict the geographical distribution and viability of natural plant populations. Understanding the molecular underpinnings of plant drought response and identifying factors involved in drought tolerance are key goals of plant abiotic stress research and crop breeding. Juenger and Verslues (2023) provide a commentary on plant–water relations and outline how the measurement of water potential in drought-related experiments can facilitate data integration and sharing across laboratories and research disciplines. Such comparisons of results between different levels of research from agronomy and ecophysiology to whole plant physiology to molecular and cell biology are needed to deepen our understanding of the mechanisms of drought tolerance and adaptation and how they may be best deployed in plant improvement. They describe how recent advances in techniques and instrumentation have made monitoring soil and plant–water status easier and more accessible to laboratories across the different fields of study in plant biology.

Two multi-author “vignette-style” articles address challenges and open questions in plant biology research related to plant abiotic stress and climate change. As a call to action for the plant science community, Eckardt et al. (2023) present examples of research aimed at improving carbon sequestering capacity and climate resilience in plants to illustrate how plant science can help to mitigate climate change and enhance food security. Verslues et al. (2023) highlight key open questions in plant abiotic stress biology as posed by 15 research groups with expertise ranging from eco-physiology to cell and molecular biology. A further set of six in-depth reviews address our understanding of the molecular bases of climate adaptation for plant improvement as well as methods and approaches for crop breeding. Napier et al. (2023) highlight progress and promising methods for identifying the key drivers of genotype-by-environment interactions, which occur when genotypes produce different phenotypic trait values in response to different environments. A companion review by Lasky et al. (2023) outlines strategies to test hypotheses based on genotype–environment associations using genetics and ecophysiology and provides recommendations for researchers seeking to learn about the molecular basis of adaptation. Bowerman et al. (2023) address how the future climate will impact growing seasons and farming systems, identifying traits and practices that are needed, and considering societal perspectives about emerging technologies for climate resilience. Cooper and Messina (2023) review methods and strategies that have been used to breed crops with improved drought resistance, focusing on the long-term improvement of temperate maize for the US Corn Belt as a case study. Charng et al. (2023) review what is known about mechanisms of plant stress memory, in particular concerning intermittent abiotic stresses, providing examples and proposing criteria for identifying components of the regulatory networks that maintain plant stress memory. Colin et al. (2023) review how plants respond and adapt to salt stress, focusing on primary cell wall biology in Arabidopsis, particularly relevant as soil salinization affects a large percentage of croplands around the world and salt tolerance is an increasingly important breeding target.

Six primary research articles in this issue address topics related to abiotic stress and climate change. Lorenzo et al. (2023) present a gene discovery pipeline in maize called BREEDIT, combining multiplex genome editing of whole gene families with crossing schemes to improve complex traits such as yield and drought tolerance. Two articles report on proteins that function in guard cells, one related to abscisic acid (ABA)-activated Ca^2+^ signaling (Tan et al., 2023) and the second describing a module that regulates microtubule disassembly (Wang et al., 2023). Both of these articles highlight the critical role of stomatal regulation in controlling water loss, which is important both to avoid dehydration and also as a key determinant of water use efficiency (amount of water lost per amount of biomass gained). Tan et al. (2023) show that multiple CYCLIC NUCLEOTIDE-GATED CHANNEL proteins act as ABA-activated Ca^2+^ channels essential for ABA regulation of cytosolic Ca2^+^ signaling and stomatal closure in Arabidopsis. Wang et al. (2023) report that another central component in ABA signaling, the SNF-Related Kinase OPEN STOMATA1 (OST1/SnRK2.6), regulates microtubule disassembly through direct phosphorylation of the plant-specific microtubule-associated protein SPIRAL1 during ABA-induced stomatal closure. Another two articles describe new aspects of the salt overly sensitive (SOS) pathway, which is well-known to be a critical determinant of salt tolerance. Fu et al. (2023) show that Clade D protein phosphatases inhibit the SOS1 Na^+^/H^+^ antiporter in the absence of salt stress but are themselves removed in response to salt to allow SOS1 to be activated by phosphorylation. Park et al. (2023) show that S-acetylation and nuclear import of SOS3/Calcineurin B-Like4 stabilizes and promotes nuclear localization of the flowering time regulator Gigantia (GI) and thus accelerates flowering during salt stress. These results provide a new molecular mechanism underlying the longstanding concept of stress escape. In this case, earlier flowering may allow plants to escape some of the detrimental effects of salt stress by completing flowering and seed production before salt-induced damage accumulates. In a similar vein, the work of Hodin et al. (2023) provides a molecular illustration of the concept of tradeoffs among stress response, growth, and metabolism. They show that a specific mutation of the tonoplast-localized transporter CLCa converts it from a 2${\text{NO}}_{3}^{-}$/1H^+^ exchanger to an anion channel. This change in CLCa activity inhibits nitrate accumulation in the vacuole and thereby increases nitrogen use efficiency. However, plants harboring the mutated CLCa are smaller than the wild-type and the authors attribute this to a reduced ability to accumulate solutes in the vacuole and consequently reduced water uptake and turgor generation. As this change limits growth even under well-watered conditions, it is likely to be more detrimental under drought conditions. Data on turgor and osmotic potentials could further quantify this interesting molecular tradeoff and how it impacts plant water status under different environmental conditions.

We hope these articles serve to inform and inspire researchers in key areas of plant biology related to climate change. We encourage authors to continue to submit their best work in plant abiotic stress biology to *The Plant Cell*. Articles in this area will be added to an online collection of articles on climate change and plant abiotic stress biology, building on the articles presented in this focus issue.
